# The market trend analysis and prospects of cancer molecular diagnostics kits

**DOI:** 10.1186/s40824-017-0111-9

**Published:** 2018-01-15

**Authors:** Ju Hwan Seo, Joon Woo Lee, Daemyeong Cho

**Affiliations:** 1 Hanyang University, 222, Wangsimni-ro, Seongdong-gu, Seoul, 04763 South Korea; 2 KISTI, 66 Heogi-ro, Dongdeamoon-gu, Seoul, 02456 South Korea

**Keywords:** Molecular diagnostics, Blood cancer, In vitro diagnostics, Market analysis

## Abstract

**Background:**

The molecular diagnostics market can be broadly divided into PCR (rt-PCR, d-PCR), NGS(Next Generation Sequencing), Microarray, FISH(Fluorescent in situ-hybridization) and other categories, based on the diagnostic technique. Also, depending on the disease being diagnosed, the market can also be divided into cancer, infectious diseases, HIV/STDs (herpes, syphilis), and women’s health issues such as breast cancer, cervical cancer, ovarian cancer, HPV(human papillomavirus), and vaginitis.

Chromosome analysis (including Fluorescent In-situ Hybridization) is one type of blood cancer diagnostic method, which involves the direct detection of individual cells with chromosomal translocation, but there have been problems of sensitivity when using this method. PCR targeting individual genes or the RT (reverse transcription)-PCR method offers outstanding sensitivity, but one drawback is the risk of false-positive reaction caused by contamination of samples, etc. Blood cancer molecular diagnostics kits allow us to overcome these shortcomings, and related products have been under development, with a focus on improving detection sensitivity, enabling multiple tests, and reducing the cost and diagnostic time.

**Results:**

Blood cancer molecular diagnostics is usually performed based on platforms such as PCR. The global market for blood cancer molecular diagnostics kits is $ 335.9 million as of 2016 and is expected to reach $ 6980 million in 2026 with an average annual growth rate of 32.9%. The market in South Korea is anticipated to grow at an average annual rate of 28.9%, from $ 3.75 million as of 2016 to $ 60.89 million in 2026.

**Conclusions:**

The Market for blood cancer molecular diagnostics kits is judged to be higher in growth possibility due to the increase in the number of cancer patients.

## Background

Molecular diagnostics (MDx) is an assay that evaluates genes, metabolic functions, drug metabolism, and disease induction based on DNA, RNA, and proteins. Molecular diagnostics is the fastest growing segment in the in vitro diagnostic market because it is the only in vitro diagnostic method that enables early diagnosis and enables preventive medical care and customized treatment. The molecular diagnostics market can be broadly divided into PCR (rt-PCR, d-PCR), NGS(Next Generation Sequencing), Microarray, FISH(Fluorescent in situ-hybridization) and other categories, based on the diagnostic technique. Also, depending on the disease being diagnosed, the market can also be divided into cancer, infectious diseases, HIV/STDs (herpes, syphilis), and women’s health issues such as breast cancer, cervical cancer, ovarian cancer, HPV (human papillomavirus), and vaginitis [1, 2].

Chromosome analysis (including Fluorescent In-situ Hybridization) is one type of blood cancer diagnostic method, which involves the direct detection of individual cells with chromosomal translocation, but there have been problems of sensitivity when using this method. PCR that targets individual genes or the RT (reverse transcription)-PCR method offers outstanding levels of sensitivity, but one drawback is that there is a risk of false-positive reaction caused by contamination of samples, etc. Molecular diagnostics kits for leukemia and other cancers have been recently developed and released on the market to overcome these shortcomings, and these kits use a quantitative polymerase chain reaction method, which enables the quantification of target RNA from even a trace amount of the sample. However, this method does pose problems in terms of cost and time, because many genes associated with cancer development must be examined to improve the accuracy of the diagnosis. In response, related products are being developed with the goal of improving the sensitivity of diagnosis, enabling multiple tests, and reducing the cost and diagnosis time.

## Methods

### Primary research

For primary research sources, we relied on the databases of the Korea Institute of Science Technology Information, past industry research services/studies, economic and demographic data, and trade and industry journals. This research was conducted to analyze the market and technology trends.

### Secondary research

For the secondary research, we performed a demand analysis for potential users of the diagnostic kit, in connection to the data from our primary research.

## Results and discussion

### Characteristics of the molecular diagnostics industry and market

Some of the characteristics of the molecular diagnostics market are that the number of patients remains at a sustained level regardless of economic fluctuations and therefore the corresponding demand for medical products and devices are also tend not to be significantly affected by economic fluctuations. This is a market that is not significantly impacted by changes in household spending, the gross national product, or economic fluctuations.

The molecular diagnostics market is an example of knowledge-driven industries that generate high added value through the input of intangible value. Once a single diagnostic technology is commercialized and becomes recognized in the world market, it can generate added value that is hundreds of thousands of times larger than the investment costs. Furthermore, the cost and time required for development and the time it takes to achieve commercialization tend to be less compared to the pharmaceutical industry, and therefore the business stability is high.The cancer molecular diagnostics kit is field with fast-paced technological progress and thus the time until a product is replaced tends to be short, which means that continued investment in research and development is required. The molecular diagnostics market is expected to grow rapidly, due to factors such as the shift in the disease management paradigm from a focus on treatment to a stronger focus on prevention through early diagnosis, the high morbidity rate of infectious diseases and various cancers, the growth of the biomarker market and the development of molecular technology, the progress in genomics and proteomics, the expanded availability of Point of Care (POC) testing, etc.

Except for a few self-diagnostic devices, the users of cancer molecular diagnostics kits are limited to professionals in medical services and these users tend to prioritize safety and reliability, which sets a high barrier to market entry.

Molecular diagnostics not only relies on foundational fields of medical science such as disease mechanism research, basic life science, and clinical medicine, but also requires organic cooperation with specialists in machinery, materials, computer and electronics especially because of the computerization of the process of diagnosis result notification between the hospital and the laboratory. It is thus a high-tech industry that has a large impact on disseminating technology to other fields.

Building on the available basic research, further research for cancer molecular diagnostics kits are currently being conducted by universities, research institutes, and bio-ventures over the long term. Since the research involving bio-information can lead to product development through outsourcing and other means, this is a field in which SMEs can successfully gain their competitiveness through technology development.

The molecular diagnostics market is an oligopoly, since a small number of global companies that possess the core technologies account for 70–80% of the market. Therefore, the market concentration is high and there is not much variation in the level of competition. South Korea is currently entirely dependent on imports for blood cancer diagnostic kits and is experiencing high technological barriers to entry [3–5].

### The size and prospects of the global market for blood cancer molecular diagnostics kits

Cancer molecular diagnostics kits belong to the outpatient diagnostic market, which is expected to grow from $ 60.25 billion in 2016 to $ 78.74 billion by 2021, with an average annual growth rate of 5.5%. Within this market, the molecular diagnostics market is expected to grow from $ 6.54 billion in 2016 to reach $ 10.12 billion in 2021, with an average annual growth rate of 9.1% [6].

Molecular diagnostics has demonstrated the highest diagnostic accuracy within the field of in vitro diagnostics and for this reason, its market is expected to have the highest growth compared to other in vitro diagnostic markets. Strong growth is anticipated in Korea, China and India, where the markets are in the growth stage, due to the increasing demand for clinical testing and analysis. Market size of the global cancer diagnosis by technology platform presents in Table 1.

**Table 1:** Market size of the global cancer diagnosis by technology platform (Unit: million $) [7]

Category	2013	2014	2015	2016	2017	2018	2019	CAGR
RT-PCR	710.5	758.9	998.2	1313.0	1727.1	2271.8	2988.3	31.5%
DNA microarray	357.1	394.4	536.1	728.7	990.4	1346.3	1829.9	35.9%
LOAC	177.9	187.0	240.7	309.8	398.7	513.2	660.5	28.7%
NGS	174.5	240.0	414.3	715.2	1234.6	2131.3	3679.2	72.6%
Multiplex conventional	92.2	98.8	131.9	176.1	235.1	313.9	419.1	33.5%
Next generation capture	68.2	76.7	124.7	202.8	329.7	536.1	871.7	62.6%
Protein microarray	18.0	20.4	29.3	42.2	60.6	87.2	125.3	43.8%
Other	25.3	31.7	38.9	47.8	58.6	71.9	88.3	22.7%
Total	1623.7	1807.9	2514.2	3535.5	5035.0	7271.7	10,662.3	42.6%

The diagnostic markets for leukemia and lymphoma, which are types of blood cancers, were estimated to be $ 117.4 million in 2013 and $ 140.2 million in 2014, and it is projected to reach $ 1.4805 billion in 2019 with an average annual growth of 60.2% (Table 2).

**Table 2:** Market size of the global cancer molecular diagnostics by cancer type (Unit: million $) [7]

Category / Year	2013	2014	2015	2016	2017	2018	2019	CAGR
Breast cancer	375.4	383.8	531.3	735.5	1018.2	1409.5	1951.2	38.4%
Colorectal cancer	346.5	391.6	531.1	720.4	977.1	1325.2	1797.4	35.6%
Cervical cancer	259.0	267.6	304.1	345.5	392.6	446.2	507.0	13.6%
Lung cancer	113.6	128.2	197.0	302.8	465.5	715.4	1099.6	53.7%
Precancer	104.5	124.9	188.3	283.7	427.7	644.6	971.5	50.7%
Prostate cancer	99.2	117.2	170.1	247.0	358.6	520.6	755.8	45.2%
Melanoma	92.0	104.8	134.6	172.8	221.8	284.8	365.7	28.4%
Leukemia	74.5	88.3	130.4	192.6	284.4	419.9	620.1	47.7%
Lymphoma	42.9	51.9	91.0	159.6	279.8	490.7	860.4	75.4%
Pancreatic cancer	26.4	32.7	47.0	67.6	97.1	139.6	200.6	43.7%
Bladder cancer	21.2	25.9	36.4	51.3	72.2	101.6	142.9	40.7%
Chest cancer	19.1	21.8	35.3	57.3	92.9	150.7	244.3	62.1%
Brain cancer	18.4	24.2	34.2	48.3	68.3	96.5	136.4	41.3%
Thyroid cancer	12.6	16.3	28.2	48.9	84.7	146.6	253.9	73.2%
Kidney cancer	11.7	15.6	27.6	49.0	86.8	153.9	272.8	77.2%
Ovarian cancer	6.7	9.5	16.4	28.3	49.0	84.6	146.1	72.7%
Stomach cancer	0.0	3.6	7.9	17.2	37.5	81.9	178.8	118.4%
Other							157.8	
Total	1623.7	1807.9	2511.1	3527.8	5014.1	7212.2	10,662.3	42.6%

The market for the molecular diagnostics of blood cancer is mainly based on PCR, and although the price is high, most accurate real-time PCR method (with more than 99% accuracy) in the one mainly used in many developed countries including Korea, while developing countries mainly use the conventional PCR (more than 90% accuracy), which is cheaper than real-time PCR. In underdeveloped countries, the molecular diagnostics market is still the early formative stages, and because of the high prices of the other options, these countries usually use Rapid PCR (60–70% accuracy). Within the field of blood cancer molecular diagnostics, the technology exhibiting the highest rate of growth is NGS [7].

The market for RT-PCR-based blood cancer diagnoses was $ 41.5 million in 2013 and $ 44.4 million in 2014, and is anticipated to reach $ 192.9 million in 2019 with an average annual growth rate of 34.1% (Table 3).

**Table 3:** The global market size for molecular diagnostics blood cancer diagnoses (Unit: million $) [7]

Category / Year		2013	2014	2015	2016	2017	2018	2019	CAGR
RT-PCR	Leukemia	35.7	37.6	47.6	60.3	76.4	96.7	122.5	26.6%
	Lymphoma	5.8	6.8	10.9	17.3	27.6	44.1	70.4	59.6%
	Sub-total	41.5	44.4	58.5	77.6	104.0	140.8	192.9	34.1%
DNA microarray	Leukemia	20.8	22.0	28.8	37.7	49.3	64.5	84.4	30.9%
	Lymphoma	10.2	11.9	20.5	35.3	60.7	104.5	179.8	72.1%
	Sub-total	31.0	33.9	49.3	73.0	110.0	169.0	264.2	50.8%
LOAC	Leukemia	3.0	3.2	4.1	5.2	6.7	8.5	10.9	27.8%
	Lymphoma								
	Sub-total	3.0	3.2	4.1	5.2	6.7	8.5	10.9	27.8%
NGS	Leukemia	15.0	25.5	44.3	76.9	133.5	231.7	402.3	73.6%
	Lymphoma	13.8	17.8	34.2	65.9	126.8	244.0	469.4	92.4%
	Sub-total	28.8	43.3	78.5	142.8	260.3	475.7	871.7	82.3%
Multiplex conventional	Leukemia								
	Lymphoma	13.1	15.4	24.0	37.3	58.1	90.4	140.8	55.7%
	Sub-total	13.1	15.4	24.0	37.3	58.1	90.4	140.8	55.7%
Blood cancer total		117.4	140.2	214.4	335.9	539.1	884.4	1480.5	60.2%

The global market for the molecular diagnostics of blood cancer is expected to grow from $ 140.2 million in 2014 to $ 1.4805 billion in 2019, achieving an average annual growth rate of 60.2%.

The market for early diagnoses and cancer molecular diagnostics kits are forecasted to grow due to factors including the increased incidence of cancer, growth of biomarker market and development of molecular technology. The global platform-based cancer diagnostic market was estimated to be $ 2.13 billion in 2015 and is projected to grow at an annual average rate of 24.8%, reaching $ 6.647 billion by 2020 (Table 4).

**Table 4:** Forecast of the Platform-based Cancer Diagnosis Market by Region (Unit: million $, %) [8]

Category	2015	2016	2017	2018	2019	2020	CAGR
North America	971.9	1215.0	1518.8	1898.7	2373.6	2967.2	25.0%
Europe	538.0	654.1	795.3	966.9	1175.5	1429.2	21.6%
Asia	406.5	521.5	669.0	858.2	1100.9	1412.3	28.3%
Other	213.5	264.9	328.8	408.0	506.2	628.2	24.1%
Total	2129.9	2655.5	3311.8	4131.7	5156.2	6436.9	24.8%

**Fig. 1:**
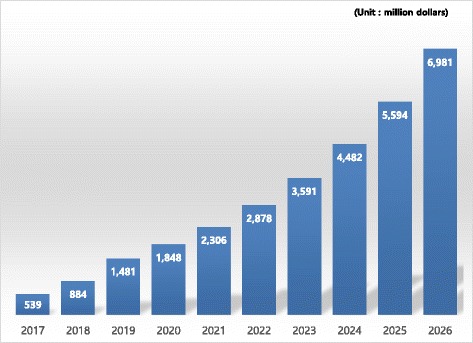
Forecasts for the global market for blood cancer molecular diagnostics kits [8]

Based on the data on the global market for molecular diagnostics of blood cancer, we performed a forecast of the platform-based cancer diagnosis market from 2020, by applying a growth rate of 24.8%. According to our results, the global market for blood cancer molecular diagnostics kits is expected to catapult from $ 335.9 million in 2016 to $ 6.980 billion in 2026, with an average annual growth rate of 32.9% [8] (Fig. 1).

### Size and prospects of the market for blood cancer molecular diagnostics kits in South Korea

**Fig. 2:**
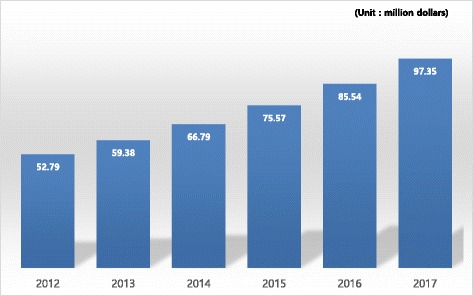
Size of the domestic molecular diagnostics market [9]

The size of the molecular diagnostics market in South Korea is expected to increase from $ 52.79 million in 2012 to $ 97.35 million in 2017, showing an annual average growth rate of 13% (Fig. 2).

The most common type of cancer in 2014 was thyroid cancer, followed in ranking by stomach cancer, colon cancer, lung cancer, breast cancer, liver cancer, and prostate cancer.

According to the Korea Central Cancer Registry published in 2016, there were 217,057 cancer cases in South Korea in 2014, among these, the most frequently occurring type was thyroid cancer, followed by stomach cancer, colon cancer, lung cancer, breast cancer, liver cancer, and prostate cancer in order of frequency [10].

Within the category of blood cancer, there were 4948 cases of Non-Hodgkin lymphoma, 2080 cases of myeloid leukemia, 1396 cases of multiple myeloma, 642 cases of lymphocytic leukemia, 140 cases of pediatric lymphoma, 251 cases of pediatric leukemia, and 5090 cases of malignant lymphoma. Within the total cancer incidence, the number of blood cancers accounted for 6.7% (Table 5).

**Table 5:** Blood cancer incidence in South Korea (Unit: Number of individuals, %) [10]

Type of Cancer		Number of Patients	Percentage
All cancers		217,057	100.00%
Blood cancer	Non-Hodgkin lymphoma	4948	2.28%
	Myeloid leukemia	2080	0.96%
	Multiple myeloma	1396	0.64%
	Lymphocytic leukemia	642	0.30%
	Pediatric lymphoma	140	0.06%
	Pediatric leukemia	251	0.12%
	Malignant lymphoma	5090	2.35%
	Blood cancer total	14,547	6.70%

As with other types of cancers, the higher the age group, the higher the prevalence of blood cancer. Analysis shows that recently, the incidence of cancer patients has steadily increased due to environmental problems and changes in eating habits which is also why molecular diagnostics technology for monitoring cancer treatment and performing prognoses is becoming more and more the important.

Unfortunately, there is no objectively reliable data regarding the South Korean market for blood cancer molecular diagnostics, and therefore our estimates were based on the percentage that cancer diagnoses occupy within the global molecular diagnostics market and more specifically, the percentage of diagnoses of blood cancer. The resulting estimate was that the size of the blood cancer molecular diagnostics market in South Korea is approximately $ 3.84 million as of 2016 (Table 6).

**Table 6:** Size of the domestic blood cancer molecular diagnostics market (Unit: million $) [7–11]

Category	Market Size		Percentage	
	2016	2017	2016	2017
Size of the molecular diagnostics market	85.54	97.35	100.0%	100.0%
Size of the cancer diagnosis market	46.11	68.34	53.9%	70.2%
Size of the blood cancer molecular diagnostics market	3.84	6.46	4.5%	6.6%

**Fig. 3:**
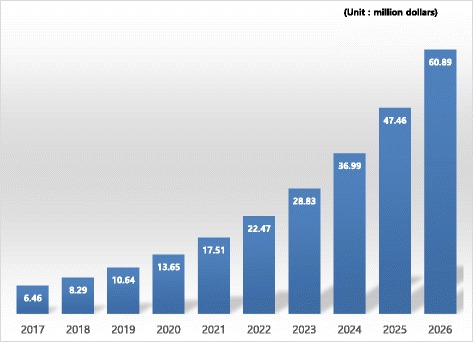
Forecast of domestic markets for blood cancer molecular diagnostics kits [8]

The platform-based cancer diagnoses market in the Asia-Pacific region is projected to growth at an average annual rate of 28.3% from 2015, and if we forecast the estimate of South Korea’s blood cancer molecular diagnostics market size at the same growth rate (28.3%) as applied in the Asia-Pacific forecast, we can project that the market will be $ 60.89 million in 2026 (Fig. 3).

### Trends in industries related to molecular diagnostics kits

As of 2013, Roche had the largest market share of the global molecular diagnostics market, comprising 25.2%. The market concentration is high, as the market share of the three top-ranked companies, namely Roche, Hologic, and Qiagen, totaled 51.2%.

Roche occupied 22% of the molecular diagnostics market in the United States as of 2015, while Cepheid, Abbott, and Themofisher respectively had shares of 14, 11, and 9%. This means that the top 4 companies comprised 56% of the market (Table 7).

**Table 7:** Molecular diagnostics device global market shares by company [3]

Company	Market Share
Roche	25.20%
Hologic	14.40%
Qiagen	11.60%
BD	9.20%
Abbott	8.40%
Cepheid	5.50%
Siemens	5.20%
Others	20.50%
Total	100.00%

Companies participating in the blood cancer diagnostics market include InVivoScribe, Asuragen. Biological Dynamics, Cancer Genetics, BlueGnome (Illumina), Cepheid, Combimatrix, Foundation One, Ipsogen, MLL, Sequenta, SkylineDx, Signature Genomics, Clarient (GE Healthcare), Insight Genetics, etc.

InVivoScribe is a global company that has provided clonality and biomarker test solutions with specializations in oncology, customized molecular diagnostics, and customized molecular medicine. This company has acquired ISO certifications (13485) and complies with cGMP (Current Good Manufacturing Practices enforced by the US Food and Drug Administration) in its factories that supply PCR, NGS-based reagents and RUO test kits. It also supplies IdentiClone® tests for genetic MRD (minimal residual disease) and somatic cell mutation and CE certified IVD (in vitro diagnostics) including LymphoTrack® and LymphoTrack® Dx analysis methods. Clinical laboratories of this company in the United States, Europe, and Japan have been internationally standardized and provides clinical tests and CRO services with CLIA, CAP, ISO15189 certifications. The kits and reagents from this company is used in around 650 medical and research institutes in 65 countries worldwide.

Asuragen, which stemmed from the University of Texas, was established in 1989 and sells molecular diagnostics products based on miRNA. In May 2013, Asuragen launched the SuraSeq 500 Clinical Cancer Panel.

Biological Dynamics was founded in 2009 as a spin-off from University of California San Diego. This company performs IgVH mutation tests for CLL and Bcr-Abl tests for CML.

Cancer Genetics Inc. develops and sells DNA-based hematology, genitourinary and HPV-related cancer diagnoses products. This company offers products for performing diagnoses with the DNA-FISH probe, CGH microarray and NGS platforms, and had a sales total of 10,771 items in 2013.

Cepheid develops, manufactures and sells genetic analysis products for clinical trials. The technical focus of this company is on the integration and automation of genetic analysis procedures including sample preparation, DNA amplification and detection.

SkylineDx is a company that branched off from the Erasmus University Medical Center (Rotterdam, Netherlands) that has developed and commercialized blood cancer diagnostic products based on microarray platforms. In September 2013 SkylineDx acquired the IP for microarray testing and Skyline diagnostics and established a new company. The featured products of this company are MM profiler and AML profiler.

Established in 2007, Sequenta Inc. is engaged in the development and marketing of clinical molecular diagnostics products. The company’s technology platform, LymphoSight, measures immune system activity by profiling T and B cell receptors. This company launched the NGS-based test ClonoSight in February 2013. ClonoSight can be used to diagnose diseases such as leukemia and lymphoma using Sequenta’s LymphoSight platform, which performs sequencing of unique DNA.

South Korea manufactures only 34% of its in vitro diagnostic reagents and is highly dependent on imports, which account for 66% of products. The top seven companies, namely Roche, Abbotte, BIOMERIEUX, BECKMAN COULTER, i-SENS, LG Life Sciences, and Seegene, were found to comprise 48% of the total market. In the field of blood cancer molecular diagnostics, the domestic market size is smaller than that of the global market, and there are not many participating companies, but the development of related products has been underway at a few companies such as Seegene and BioSewoom.

## Conclusion

The cancer diagnoses market is projected to grow due to technological advances, an increase in cancer patients, and a surge in the elderly population. Furthermore, the molecular diagnostics market is expected to grow significantly as it demonstrates is ability to perform genetic tests that were previously unavailable and replaces the existing clinical chemistry, immunology, and microbiological tests.

Cancer molecular diagnostics is led by global companies such as Roche, Qiagens and Becton Abbott laboratories. Companies competing in the market for blood cancer molecular diagnostics kits include InVivoScribe, Asuragen. Biological Dynamics, and Cancer Genetics, and it appears that the market competition is not likely to change significantly because the technology barriers remain high.

Seegene and BioSewoom are companies that participate in South Korea’s molecular diagnostics kit market, but Seegene is predominantly focused on diagnosing infectious diseases and BioSewoom does sell blood cancer molecular diagnostics kits, but the sales performance in this category is lagging. The majority of the blood cancer molecular diagnostics kits used in South Korea is imports.

The global market for next-generation cancer diagnoses is projected to follow an annual average growth rate of 42.6%, expanding from $ 1.8 billion in 2014 to $ 10.629 billion in 2019. The size of the blood cancer molecular diagnostics market is expected to increase from $ 539 million in 2017 to $ 6.980 billion in 2026 with an annual average growth rate of 32.9%. South Korea’s market for blood cancer molecular diagnostics was $ 3.84 million in 2016, which is not large, but it is anticipated to grow at a robust rate of around 32.2%, similar to the global growth rate, and therefore it is deemed an attractive market for blood cancer molecular diagnostics in terms of growth potential.

In conclusion, blood cancer molecular diagnostics kits represent the leading achievement in molecular diagnostics technology with the highest accuracy among available in vitro diagnostic devices, and we anticipate that the demand will continue to increase due to the rising number of cancer patients and the shift of priorities in the medical field from treatment to prevention.

## Abbreviations

FISH: Fluorescent in situ-hybridization
HPV: Human papilloma virus
NGS: Next generation sequencing
PCR: Polymerase chain reaction
POC: Point of care
RT-PCR: Reverse transcription polymerase chain reaction
